# Beyond the Single Isolate: Leveraging Plant-Associated Microbial Communities for Crop Resilience

**DOI:** 10.3390/microorganisms14020456

**Published:** 2026-02-13

**Authors:** Ashish Kumar Sarker, Karishma D. Kuar, Esha Kuriakose, C. Oliver Morton, Colin M. Stack, Michelle C. Moffitt

**Affiliations:** 1 School of Science, Western Sydney University, Campbelltown, NSW 2560, Australiao.morton@westernsydney.edu.au (C.O.M.); 2 Department of Pharmacy, Pabna University of Science and Technology, Pabna 6600, Bangladesh

**Keywords:** biocontrol agents, biofertilisers, pesticides, seed coating, synthetic microbial communities

## Abstract

The future of sustainable agriculture will require practical microbial solutions that reduce chemical inputs while maintaining productivity. While existing literature reviews focus on laboratory science, they rarely address the practicalities of farm implementation. Low rates of adoption suggest a translational gap. This review translates current scientific insights for the relevant end user (farmers). Pesticides and fertilisers disrupt naturally occurring microbial communities that maintain plant health and resilience. Applications of beneficial microbes to restore plant health or improve productivity currently employ single-strain inoculants. The targeted application of a consortium of multiple microorganisms, a “synthetic community” (SynCom), including biocontrol agents, biostimulants and biofertilisers, is superior. The “SynCom” approach could be considered the Swiss army knife of sustainable agriculture, with each member of the community performing overlapping functions. While SymComs have shown success in laboratory and greenhouse trials, field reliability has been inconsistent, either due to variability in production or stability issues in the field. The future of sustainable agriculture will require greater collaboration between scientists and farmers at a local level, specifically, the application of microbes from local soils that are adapted to local environmental conditions, investment in monitoring successes and failures, and application via seed coating using currently available infrastructure.

## 1. Introduction

Every year, farmers face the same seemingly impossible financial balancing act, one that would give the management of any large business nightmares. They commit thousands to millions of dollars upfront in unpredictable input costs (fertilisers, seeds, chemicals, and fuel) all without knowing what prices their crops will fetch at harvest. While agriculture has historically kept pace with global demand, we are now at a critical juncture. The Green Revolution dramatically increased global food production; however, it has come at a hidden cost [1]. The pursuit of higher yields has been inextricably linked to an unsustainable reliance on synthetic agrichemicals, with unintended consequences for long-term soil health [2]. This has created a vicious cycle of dependency, in which declining soil fertility necessitates increased chemical inputs, eroding biological resilience and farm profitability. At the same time, urbanisation has reduced arable land availability, while climate uncertainty has undermined the predictability on which traditional farming systems relied [1,3].

Farmers have responded by rapidly adopting technological and agronomic innovations, including precision agriculture tools, improved seed varieties, and conservation practices such as crop rotation and resting paddocks. However, despite these advances, modern agriculture remains heavily dependent on synthetic fertilisers and broad-spectrum pesticides [4]. Ironically, farmers are now more dependent than ever on these expensive chemical inputs, and eroding profitability, with increased costs being passed to the consumer [5,6]. It can take decades for new developments in agriculture to be realised; however, investment has been steadily decreasing in research and development [6].

A growing body of evidence indicates that pesticides can disrupt native beneficial plant-associated microbiomes, altering their abundance, diversity, and functional capacity both below the ground, the rhizosphere, and above the ground in plant tissues, the phyllosphere [7]. These effects parallel the disruption of the human microbiome by antibiotics, which is increasingly recognised as central to human health [7,8,9]. The human microbiome can be partially restored through the use of probiotics or in extreme cases through fecal microbial transplants [10]. In agriculture, microbial-based interventions, including but not limited to biocontrol agents (BCAs), mycorrhizal fungi, and nitrogen-fixing bacteria have been employed as single inoculants to improve plant health and reduce chemical inputs [11,12].

More recently, the development of synthetic microbial communities (SynComs) has emerged as a promising strategy to restore and enhance beneficial plant-associated and soil microbial function. By rationally combining complementary microbial strains, SynComs can be tailored to local conditions to improve crop resilience and promote long-term soil recovery [13,14,15]. The key to the success of this tool is the synergistic and functional integration of microbes [16]. Together these approaches offer a pathway towards reducing agriculture’s dependence on synthetic inputs and addressing the sustainability challenges facing modern food production [17].

Despite growing interest in beneficial soil microbes, a gap between research and farm practice remains. While most scientific reviews focus on mechanisms and experimental work, there needs to be more attention to implementation challenges, including cost, ease of application, reliability under field conditions, and integration with existing practices.

This review aims to bridge the research–practice gap by identifying the need for SynComs, evaluating current research and identifying opportunities for field implementation. We translate the relevant microbial science for a broader audience, including agricultural decision-makers. We highlight both the limitations of current agricultural practices and opportunities SynComs offer for sustainable agriculture through improved soil and plant health, nutrient cycling and disease suppression. We acknowledge that these technologies are still in their infancy and discuss what they can and cannot do. Evidence suggests that SynComs can be adopted to help build resilient and sustainable cropping systems, in the face of climate uncertainty and input cost pressures. However, involving key decision-makers early will be crucial if technologies such as SynComs are to become an agricultural reality. Like the utility farmers have come to know and appreciate in a tool such as a Swiss army knife, SynComs represent complementary microbes working together. They can be combined on a single seed and represent a single purchasing decision.

## 2. Current Practices Alter Soils and Plant-Associated Microbiomes and Their Beneficial Functions

**Figure 1:**
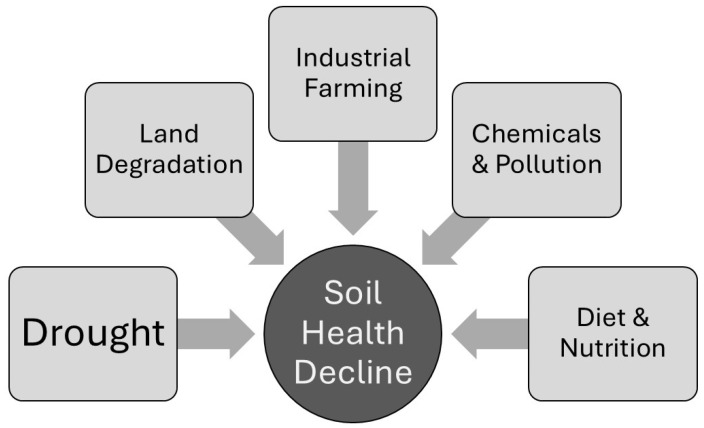
Factors contributing to global soil degradation. Land clearance and extreme weather events lead to land degradation directly contributing to reduced soil health. In turn this can lead to drought because land degradation reduces soil water retention. Intensive agriculture requires large inputs of fertilizers and industrial machinery which further lead to land degradation and loss of soil biodiversity. Human dietary choices lead to crop monocultures and intensive animal production that cause reduced soil quality. Overall, industrial farming and pollution are key agents of soil decay that also disrupt soil microbial populations (based on information from the UN Environment Programme https://www.unep.org/news-and-stories/story/five-reasons-why-soil-health-declining-worldwide, accessed on 17 December 2025).

Over the past decades, the level of organic matter in soils has dropped significantly, while soil microbial diversity has decreased or changed in abundance [18]. While many farmers are aware of the environmental and human impacts of agrichemicals, some may not fully realise that in their quest for increased yields, the annual addition of synthetic inputs such as pesticides can inadvertently degrade soil health through disruption of the soil’s microbiome (Figure 1). This destabilisation can be linked to the extensive use of pesticides, defined here as a substance that is used to control an organism that causes damage in agriculture and includes herbicides, insecticides, fungicides and antibacterials [7]. Rather than indiscriminately spraying, many farmers have relied on the targeted application of these agents through their incorporation as seed coatings. However, these broad-spectrum/untargeted fungicides and antibiotics can still impact beneficial microbes in the soil adjacent to planted seeds, whose role it is to decompose organic matter and recycle soil carbon. Furthermore, the use of an agent with a single mode of action can lead to the development of pathogen resistance [19]. Significantly, these microbes can also play an essential role in maintaining soil structure. Deteriorated soils display poor water infiltration, resulting in greater compaction and erosion.

Frequent use of agrichemicals can also impact beneficial plant-associated microbiomes, changing their abundance, diversity, composition, and functionality (Table 1) [7]. The rhizosphere encompasses the below-ground plant-associated microorganisms, including those associated with the root surface (ectorhizosphere) and those that colonise within the root tissue (endorhizoshpere) [20]. Some rhizosphere organisms can help train plant defence mechanisms, referred to as induced systemic resistance, enabling plants to resist above-ground infections. Rhizosphere microbes are also able to promote plant growth, enhance nutrient absorption, water infiltration and, more broadly, are important in providing the plant with nitrogen [20,21,22]. Similarly, the phyllosphere is host to diverse epiphytic (on plant surfaces) and endophytic (within plant tissues) microbial communities, which play a crucial role in plant physiology, and growth [23]. Beneficial microbes are recruited to this community via the leaf structure and chemicals produced by leaf cells. Evidence suggests that these above-ground microbial communities are not only impacted by spraying but can also be altered by the systemic uptake of soil root-applied pesticides [24].

Even though they are designed to target specific pests, pesticides interact with microorganisms through several interconnected mechanisms (Table 1) [7]. Direct effects involving interaction with microbial metabolism, alteration of cellular structure, hampering growth, and inducing oxidative stress, lead to microbial dysfunction or death [7]. This changes the abundance of susceptible beneficial microbes and can subsequently weaken plant natural defences to environmental/abiotic stresses such as drought, temperature and salinity, and disease or pest attack (biotic or biological stress). Decreasing numbers of certain bacterial species can also impact nutrient cycling. Nitrogen cycling, performed primarily by microbes, is considered an indicator of soil health [25,26]. However, agriculturally applied fungicides and antibiotics have been observed to change the abundance of key microbial populations, such as *Rhizobium* [27,28,29,30]. In contrast, carbon cycling is performed by non-specialist organisms through the breakdown of complex molecules including agrichemicals. A study of 20 pesticides in different soil types found that in response to some pesticides, carbon decomposition pathways can be stimulated, potentially resulting in an alteration of the microbial population towards decomposers, although effects are dependent on pesticide and soil type [31].

Indirectly, even if microbes are not killed by pesticides, they may still impact the microbial community structure (Table 1) [30,32,33]. Often the beneficial microbes work together as a network, analogous to a “farm to consumer” supply chain. Each microbe is connected with others around it, depending on another’s product or byproduct for nutrition/energy for example [34]. By working together, these microbes create a consortium of complementary metabolic pathways, invisible partnerships and dependencies, that enable the community to perform functions that no single organism could perform alone. However, when one species within the network is susceptible to an agrichemical treatment, it has the effect of causing the network to breakdown, similar to when one step in a consumer supply chain is disrupted [30,32,33]. This can leave niche gaps, space that may become occupied by other microbes that may not be beneficial [35,36].

Furthermore, the over-reliance on single mode of action antifungal and antibacterial agents can lead to the development of resistance within agricultural pathogens, which has occurred with the antibiotic streptomycin and the azole antifungals. Significantly, these same agents are also used to treat human pathogens [19,37]. Our understanding of the function of microbes working together is still in its infancy, and so the destructive impact of these chemicals on communities may be underestimated in pesticide risk assessments, which typically emphasise acute pesticide effects rather than overall ecological consequences.

**Table 1:** Examples of ways in which agrichemicals can impact plant-associated beneficial microbes.

Pesticide Function	Example Chemicals	Variations of the Beneficial Microbial Community	Target	References
Fungicide	Difenoconasole Hymexazol Iprodione Dazomet Triadimenol, fluopyram, and penthiopyrad Carbendazim	Changes in abundance of target (fungal) and non-target (bacterial) organisms Decreased co-occurrence network. Changes to degradation and nitrogen cycling	Soil Pepper Cucumber Watermelon Tabacco	[30,35,38,39,40,41]
Antibacterial	Tetracycline Oxytetracycline and penicillin Streptomycin and kasugamycin	Bacterial diversity decreased, treatment-dependent Increase in streptomycin resistance compared with kasugamycin	Wheat Grapefruit Apple	[36,42,43]
Insecticide	Lindane Chlorpyrifos and quinalphos Clothianidin Deltamethrin Triflumuron and fenoxaprop-P-ethyl	Decreased abundance of bacterial and fungal endophytes Decreased arginine ammonification ability (N-cycle) of rhizospheric microbes	Rice Peanut Sugarcane Pepper Chinese cabbage Soybean	[44,45,46,47,48,49]
Herbicide	Halosulfuron methyl Diclofop-methyl Imazethapyr Cypermethrin S-metolachlor Glyphosate R-Dichlorprop	Changes in abundance of bacteria in the phyllosphere and rhizosphere, chemical dependent, including *Rhizobium* Root colonisation and spore viability of arbuscular mycorrhizal fungi (AMF) decreased	Sugarcane Rice Arabidopsis Wheat Grass	[50,51,52,53,54,55]

## 3. Single-Strain Microbial Inoculants

The use of single-strain inoculants, referred to as bioinoculants, in broadacre and protective (greenhouse or controlled environment) cropping has a long history with varied results (for reviews see [22,56]). While many early successful trials were carried out under controlled conditions (greenhouse settings), their replication under real-world or open-cropping conditions produced inconsistent results [57]. Despite being commercially available, their uptake has been slow. There are several reasons for this: a lack of regulatory pressure on sustainability, scepticism by farmers, and historically the cheap and plentiful supply of synthetic inputs [58]. Inconsistencies in commercially available products have also been problematic with little understanding of the causes of failures [59]. However, faced with increased costs, shrinking margins and a renewed regulatory focus to reduce synthetic inputs, under initiatives such as the European Union’s Green Deal, microbial inoculants are now being explored with renewed enthusiasm, spurred on by advancements in seed-coating technologies [60]. As seed-coating technologies evolved commercially, they started to integrate not only microbes but microbial products as well for use in disease suppression or biofertilisation [61].

### 3.1. Microbial Seed Coating as Biofertilisers and Biostimulants

To help move agriculture towards a more sustainable setting, scientists started searching for soil microorganisms, with the goal of finding microorganisms with specialised functionalities [62]. This bioprospecting exercise used a reductionist approach and led to the identification of “elite” or “specialist” strains (e.g., nitrogen fixation or phosphate solubilisation) [26,63]. These microbes or single-strain inoculants can be mass produced and incorporated, often through foliar sprays, soil drenching (more applicable in protected cropping settings) or directly applied through microencapsulation onto seeds ready for sowing [64,65]. Application directly into a seed coating is likely to be the most practical for widespread use in broadacre settings given the sheer scale. When using coated seeds, farmers do not require any new equipment or need to change their practices, as implementation aligns perfectly with what they already do. Ideally, once established, seed coatings break down, releasing their microbial package into the soil adjacent to the seed.

Early inoculants of plant beneficial microorganisms include bacteria (referred to as plant growth-promoting rhizobia, PGPR) such as *Rhizobium* used to inoculate legumes like soybean, as well as arbuscular mycorrhizal fungi and other fungi, such as *Trichoderma* spp. [64]. The incorporation of this technology has brought a higher level of sophistication to agriculture (for reviews see [66,67]).

An important direct benefit is the enhancement of nutrient availability [62]. For example, there are a number of nitrogen-fixing microbes such as *Rhizobium* and *Azotobacter* that can be added to coatings; once established, these bacteria can convert atmospheric nitrogen (N_2_ gas) into usable forms, e.g., ammonia (NH_3_) and ammonium (NH_4_^+^), that promote plant growth [26]. Inoculation of legumes represents the first successful use of single inoculants in agriculture, which utilises strains of *Rhizobium*, *Bradyrhizobium*, or *Ensifer* (formerly *Sinorhizobium*) to establish symbiotically in the root nodule of the legume [58,68]. The success of these inoculants can be variable, depending on interactions with native microbes or soil conditions [69]. For other non-leguminous plant crops, free-living bacteria such as *Azotobacter* utilise carbon sources secreted from the plant roots and fixed nitrogen, fertilising the surrounding soil with ammonia or ammonium [68].

Next to nitrogen, phosphorus (P) is nutritionally the second most important element, key to plant vitality and disease resistance. Although abundant in soils, it is predominantly found in an immobilised/insoluble form, phosphate, and is inaccessible to plants [70], requiring farmers to apply it in the form of liquid phosphate fertilisers. Initially absorbed by plants, it quickly becomes chemically bound to soil minerals and organic matter, becoming locked up or “fixed” and unavailable for root uptake. In terms of sustainability and impact of farm profitability, P is a finite resource, its synthesis is energy-intensive, and its cost has seen significant increases in the past decade. Furthermore, its excessive addition to soils can result in adverse environmental impacts, such as eutrophication [71]. As mentioned above, because of farming practices, there are already large quantities of unusable P in agricultural soils. Interestingly, some microorganisms naturally possess the ability to cycle and make this nutrient available. These microbes, referred to as Phosphate Solubilising Microorganisms (PSMs), include bacteria such as *Bacillus* and *Pseudomonas* spp., as well as fungi such as *Aspergillus* and *Penicillium* spp. (for reviews see [72,73]). Although these organisms are ubiquitous, their presence/density in soils can show significant variation, particularly in response to the addition of fertilisers (for reviews see [71,74]). There are several mechanisms utilised by PSMs in the solubilisation of P, two of the most well studied are: (1) some PSMs produce organic acids, which act to lower the soil’s acidity causing it to resolubilise and (2) some microbes release extracellular enzymes such as phosphatases and phytases that cleave phosphate groups from organic matter, unlocking them for absorption [75].

An important indirect function of some single-strain inoculants is to act as biostimulants, enhancing seed germination, supporting plant health and vitality and resilience to stressors (for review see [76]). This can be protection from pathogens or environmental stressors such as drought and salinity [77]. The mechanism of biostimulation is via the production of plant hormones, enzymes, and osmoregulators (see review [68,78]. These can be in the form of water-soluble compounds or volatiles. In addition to enhancing the growth of plants, biostimulants in the field can also change the community of microbes within the surrounding rhizosphere, adding to its beneficial effects [79].

The failure of some single-strain microbial inoculants to establish and colonise is clearly a hurdle to overcome for wider implementation to occur [80]. The impact(s) of environmental conditions such as soil type, moisture, pH, temperature, as well as competition from resident microbes can affect survival [57]. These variables can be difficult to predict from laboratory screenings and explain inconsistent field results.

### 3.2. Microbial Inoculants for Biological Control

The issues with toxicity and off-target effects of many pesticides have created the need for alternative measures to control plant pathogens. A good example was the move to ban the soil fumigant methyl bromide [81]. This was used to eradicate a variety of crop pests prior to planting, including plant-parasitic nematodes. Its removal meant the possibility of the return of previously well-controlled plant pests, leading to extensive research into alternatives including solarisation and biological control [81,82].

**Figure 2:**
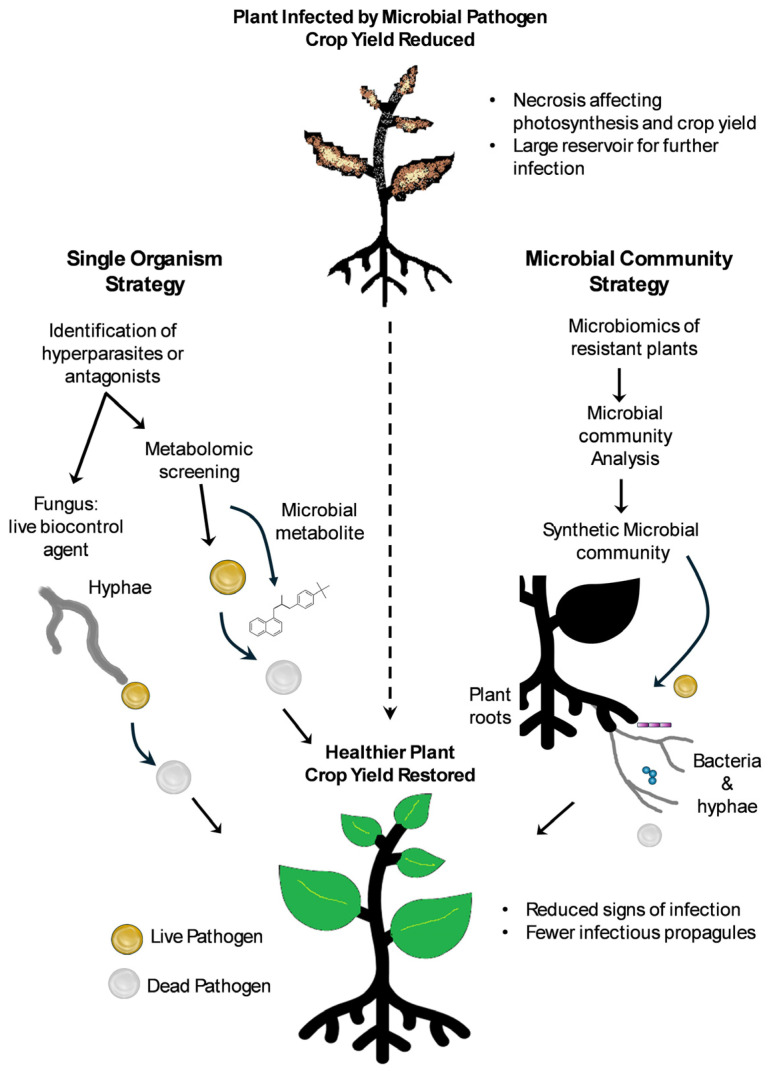
Biological control strategies. Potential biocontrol agents can be identified through culture-based methods to isolate hyperparasites or antagonists. These can be screened for use as live biocontrol agents or for metabolites which are bioactive against the pathogen. Microbial community analysis can be used to identify biocontrol agents that can be used in combination as a synthetic community.

Biological control is defined as the use of a natural antagonist, predator or parasite, that is used to control the population of a target organism that is considered a pest [83]. Biological control of plant-parasitic nematodes was first derived from the observation that some soils suppressed nematode infections [84]. It was found that certain soils contained fungi that infected the nematode egg masses, killing the developing nematodes, and thereby reducing the crop losses due to nematode infection [82]. One of the fungi that was isolated was *Pochonia chlamydosporia*; this was selected as a biological control agent because it could be cultured and formed stable spores that could be stored long-term prior to on-farm application [85,86]. Further study of the fungus revealed it displayed host specificity, with particular isolates being adapted to the plant-parasitic nematodes from which they were isolated, meaning that use of a single isolate would not be successful against all plant-parasitic nematodes [87,88]. This reveals that it is important to study biological control agents and test them rigorously before being integrated into any pest management strategy. This can prevent disasters that have marred the reputation of biological control, such as the introduction of cane toads in Australia [89]. There are three principal biological control strategies: use of antagonistic organisms to control a pest, use of molecules derived from an antagonistic organism to control a pest, or use of microbial communities to control a pest (Figure 2).

The most successful agricultural example of a microbial biological control agent (BCA) is *Bacillus thuringiensis* (*Bt*). Unlike other BCAs, *B. thuringiensis* is not a parasite or pathogen. This bacterium produces a toxin that, once ingested, targets the feeding insect’s gut cells, causing them to swell and burst, leading to gut paralysis and death [90]. Historically, this spore-forming bacterium was grown in culture and produced as a formulation applied to crops. However, given its mode of action (parasporal crystal protein), the toxin itself was produced and applied directly as a pesticide, bypassing the need for the microbe. The *cry* genes encoding the toxin have subsequently been cloned and incorporated directly into plants, enabling genetically modified crops to be intrinsically resistant without the need for pesticide application [90]. Other bacteria belonging to the genera *Bacillus* (*Bacillus amyloliquefaciens* and *B. subtilis*) and *Pseudomonas* (*Pseudomonas chlororaphis*) are frequently used as BCAs [91]. When introduced into soils these microbes produce molecules such as lytic enzymes, antibiotics, and other metabolites that are toxic to microbial pathogens.

Another example of biological control is the use of fungi to control fungal pathogens, such as, for example, hyperparasitism, where a parasite has its own parasite. *Trichoderma harzianum* is a well-established BCA against plant pathogens such as *Fusarium solani* and *Rhizoctonia solani* [92]. *Trichoderma aggressivum* is a mould that infects cultivated mushrooms (*Agaricus bisporus*), causing a condition called green mould [93]. This *Trichoderma* sp. has also been studied to determine its potential as a BCA against plant-pathogenic fungi [94].

Despite the early setbacks there are now many commercially available biological control agents (Table 2). The mechanisms underlying biological control are often inherently multidimensional, encompassing an array of interconnected strategies, including antibiotic production (antibiosis), competition for critical nutritional substrates and ecological niches, mycoparasitic interactions characterised by direct fungal antagonism, and the induction of systemic host resistance mechanisms [95]. All these mechanisms can collectively synergise to establish a robust defence against potential pathogenic invasions. For example, *Bacillus subtilis* GB03 utilises both antibiosis and competitive mechanisms to suppress the growth of *Fusarium* spp., *Pythium* spp., and *Rhizoctonia* spp. that cause wilt disease in cotton [96,97]. Alternatively, small numbers of individual BCAs can be combined, which are referred to as consortia-based biofertilisers. Here, strains are chosen because they individually display beneficial effects rather than their interaction(s) with each other (for reviews see [98,99]). They are combined in the hope that they will work well together. While generally effective, often outperforming single-strain products, their effectiveness can be highly variable depending on soil conditions and the microbes already present in soil [100].

While BCAs are often marketed as having a lower risk of resistance compared to synthetic antimicrobials, it is important to note that they are not immune to the selection pressures placed on pathogens to evolve to overcome a given intervention [57]. For example, if the BCA’s inhibitory effect is based on a specific metabolite or toxic byproduct, the pathogen could adapt by detoxifying this molecule. The limitations of BCAs such as unstable performance in the field and narrow-spectrum capability need to be considered before they are used [95,101,102]. Furthermore, the potential for strain attenuation, where the BCA loses its effectiveness due to repeated culturing or genetic drift, is another key consideration that requires monitoring [103,104]. As with any integrated pest management plan, it is essential to implement rotational BCA strategies; this moving target approach combined with crop rotation would lessen the likelihood of resistance emerging. One potential alternative could be to isolate key metabolites from BCAs (biopesticides) that inhibit the growth of the target species [90].

Biopesticides are potentially less harmful to the environment than conventional synthetic pesticides because they already exist in the environment [105]. The strategy of identifying biopesticides is to test extracts from a potential BCA, e.g., entomopathogenic fungi or soil fungi, and screen them for effectiveness against plant pathogens [106,107]. This overlaps with early strategies that were employed to identify novel antibiotics for human pathogens, such as streptomycin, which were identified by screening environmental organisms for novel bioactive compounds [108].

**Table 2:** Examples of commercially available BCAs.

Biocontrol Agents	Crops Protected	Target Disease	Brand Name	Mechanism(s)	Target Phytopathogen	References
**Fungi**						
*Coniothyrium minitans* CON/M/91-08	Carrot, cabbage, potato, and celery	Rot, and white mold	Contans^®^ WG and Intercept	Mycoparasitism	*Sclerotinia sclerotiorum* and *S. minor*	[11,109]
*Gliocladium catenulatum* JI446/*Clonostachys rosea*	Vegetables, turf, herbs, spices, ornamentals, and trees	Grey mould	Prima stop, soil guard	Competition, mycoparasitism, and antibiosis	*Soil-borne pathogens*	[11,110]
*Phlebiopsis gigantea*	Conifer trees	Root and butt rot	Rotstop	Competition	*Heterobasidion annosum*	[12,111]
*Pseudozyma flocculosa*	Grapevines, barley, apple, wheat, roses, and vegetables	Powdery mildew	Sporodex and Sporodex L.	Parasitism	*Erysiphe*, *Microsphaera, Uncinula*, and *Podosphaera*	[112,113]
*T. album*	Tomato	Tomato wilt	Bio Zeid^®^	Antagonism	*F. oxysporum*	[114]
*T. harzianum* T22	Greenhouse nurseries	Root diseases	Root Shield^®^ and Trianum-P	Competition, mycoparasitism, and antibiosis	*Soil-borne pathogens*	[11,115]
*T. harzianum* T39	Nearly all the food crops	Rot and blight	Trichodex	Mycoparasitism	*B. cinerea*	[11]
**Bacteria**						
*Bacillus subtilis* QST 713/*B. amyloliquefaciens*	Cherries, potatoes, cucurbits, grapes, tomatoes, peppers, and walnuts	Rots, clubroot, yellow rust, and blights	Serenade	Resistance stimulated by antibiosis	*Foliar pathogens* and *Pythium*	[116]
*B. megaterium*	Tomato	Tomato wilt	Bio Arc^®^	Antagonism	*F. oxysporum*	[114]
*B. subtilis* GB03	Crop seeds (barley, cotton, wheat, peanuts, and beans)	Cotton wilts	Kodiak^®^, Companion	Antibiosis and competition	*Fusarium*, *Aspergillus*, *Pythium* and *Rhizoctonia*	[96,97]
*B. pumilus* GB34	Soybean	Root diseases	GB34 Concentrate	Antagonism and competition	*Rhizoctonia* and *Fusarium*	[11,39]
*Streptomyces griseoviridis*	Cereals (wheat, barley, rice), cucumbers, soybean, and other crops	Head blight, rot, and wilt	Mycostop^®^	Antibiosis and competition	*Fusarium*, *Pythium* and *Rhizoctonia*, *oomycetes*, and *bacteria*	[12]

## 4. Synthetic Microbial Communities: A Swiss Army Knife Approach

As indicated earlier, the use of single inoculants potentially represents a single point of failure. In some situations, this inherent narrow functionality/susceptibility to environmental stressors can be overcome. Controlled settings such as protected cropping are perfect examples of where these solutions are applicable [117]. However, in complex soil environments such as those in open farm or broadacre settings, environmental vulnerabilities can be amplified, as reflected in their well-documented inconsistent field performances [57].

Laboratory success does not guarantee effectiveness under field conditions. Farmers need a robust/dependable solution that can ensure yields before reducing their dependence on chemical-based inputs. While single inoculants have their place, farmers need something that mirrors natural microbial interactions. Plant–microbe interactions are inherently community-level occurrences where novel outcomes emerge from complex interaction of microbial networks rather than individual species [118,119].

The advent of high-throughput sequencing coupled with decreased sequencing costs has truly revolutionised our understanding of plant-associated microbiomes. Previously invisible, due to many of these microbes being unculturable, these new technologies have revealed an extraordinary level of complexity. Critically, research has revealed that these communities are not merely random groupings/assemblages of microbes, rather they have evolved over time to become highly organised and specialised, shaped by co-evolution, soil type, plant genotype, and climatic conditions [34,120,121]. As mentioned previously, plant-associated communities have a profound influence on plant health, development, and stress tolerances [118,122,123]. Plants actively recruit specific microbes using plant root exudates and signaling molecules. However, this raises a key question: when beneficial microbes and their community networks are impacted by agricultural practices, how do we rebuild these communities and, importantly, restore the essential functions they provide?

### 4.1. What Are Synthetic Microbial Communities?

Synthetic microbial communities (SynComs) represent a conceptual advance beyond the single-strain inoculants described earlier. Shifting from a single target/function to a broader systems level/holistic approach [16]. These microbial assemblages combine the beneficial properties and stability required for agricultural applications [124,125]. These can include artificially designed consortia, typically composed of multiple diverse species with complementary functions. Individual strains can be isolated from native soils, specific to a region and/or crop type, grown and tested in laboratories for their plant biostimulation, biofertilisation and/or biocontrol activities [126,127]. Genome sequence information has been and will continue to be critical to the design of these consortia, allowing for the inclusion of specific strains with the desired functionalities [128]. Critically, these combinations include complementary and emergent properties, mimicking those of natural soil and plant microbiome networks [129]. As with single strain inoculants, SynComs can be most easily incorporated directly onto seeds using microencapsulation technologies. Once in soil, they are positioned optimally to shape early microbiome assembly and subsequent root colonisation [130,131]. SynComs represent a transformative opportunity, a powerful tool that combines nature’s own microorganisms with scientific knowledge gained from molecular biology and bioinformatics to drive long-term ecosystem resilience and reduce the excessive use of agrichemicals [132]. A significant benefit of SynComs is that they offer multiple functional layers, a form of backup/redundancy ensuring greater stability. If a particular microbe within the group does not grow, and its role, for example, is nitrogen fixation, there will be another available within the group to replace its function [14]. SynComs can be tailored for specific crops, localised environmental soil, and climatic conditions [77,126].

### 4.2. Evidenced Design and Assembly Strategies

SynComs can be developed from a range of sources, including immune stimulating communities isolated from healthy plants challenged with a pathogen [133], pesticide-free communities, native counterparts, or stress-acclimatised hosts such as desert plants [126,127]. There are several factors to consider when designing a SynCom: which microorganisms need to be included, and at what ratios, and the desired outcome depending on environmental conditions, pathogen or pest, and crop type [16]. Research suggests that SynComs composed of fewer strains are more beneficial than those consisting of more strains, likely due to the inhibitory effects some individual strains might have on others [127,134]. This principle is supported by studies in banana plants, where consortia consisting of fewer isolates (11 or 3), were superior to more complex consortia (44 strains), with some isolates in the larger consortia exhibiting inhibitory effects towards other beneficials [135].

There are two primary approaches taken to SynCom design: top-down and bottom-up, both of which have their potential strengths and weaknesses (see Table 3). Top-down favours starting with a natural community. This could be from a particular crop type (wheat or maize), geographical location, high-yielding variety or location known to be farmed organically. This assemblage (50–200 species but can be more) is then simplified down to a subset of key species (10–30 species). This reductionist approach allows scientists to understand how specific microbes contribute to community performance and structure [136]. The goal is to design a minimum or model microbiome that delivers the required functional benefits while retaining stability and reproducibility. In contrast, the bottom-up approach involves the building of communities from known beneficial microorganisms (3–10 but can be up to 15 species); these can be natural or can potentially include genetically engineered strains. Bottom-up assembly is based on the functional traits required by a community, determined using sequencing and bioinformatics, which may not naturally occur together [137]. Individual organisms can also be selected based on their proven abilities in the laboratory, e.g.,for their ability to provide nitrogen to the plant, sequester iron or solubilise phosphorus [128]. These strains are then assembled into a community containing complementary functionalities [132].

In high-value controlled environments such as protected cropping, SynComs can be applied directly to the rhizosphere as drenches to protect against soil pathogens or as sprays in phyllosphere applications to target foliar pathogens [134,135]. The long-term dynamics and multi-generational effects following the application of SynComs remains unclear. However, some evidence does suggest that phyllosphere-applied SynComs improve plant health and productivity, supported by increased beneficial microbial diversity and interactions, even two years after application of the SynCom [138]. This indicates that colonisation of the host by the SynCom can become stable, with long-term benefits to the plant and potentially reducing the need for ongoing application. However, despite the successes in these approaches, we propose that more investigations into seed-coating techniques should be explored for open cropping, as handling of large quantities of liquid cultures of SynComs will be more problematic than working with smaller quantities of seed-coating materials.

## 5. The Critical Gap: Laboratory vs. Field Performance

Although SynComs hold immense promise, a fundamental challenge emerges when translating laboratory research to field applications (for review see [139]). SynComs identified as most beneficial under controlled conditions such as greenhouse experiments are not necessarily optimal under field conditions, highlighting the need for scientists to work directly with farming communities to develop more robust and adaptable field-specific consortia [126]. The development of consortia is inherently crop-specific and must be designed based on the requirements of the crop for nutrients, abiotic stresses, and target pathogen or pest, requiring extensive up-front research and testing under a range of environmental conditions [140]. Geographic location is another key consideration in terms of understanding the impact/dynamics of introducing non-native SynCom members into new areas [141]. Would new microbes integrate, or would they be out-competed by the resident native microbial community? To answer this, Jiang et al. (2023) recently investigated the integration of a native SynCom versus a commercial plant-growth-promoting rhizobacteria inoculant [142]. The same authors demonstrated, what they referred to as, a “home-field advantage” for native SynCom microbes in an open-field trial. This complexity currently inhibits broad-scale implementation in the field. Farmers need to understand that improvement of soil conditions may occur slowly over many years and may not yield the immediate improvement as expected from chemical treatments alone. We propose not to completely replace fertilisers or pesticides but rather supplement them with careful monitoring for success and the need for augmentation.

Increased short-term investment in industries and facilities that can establish a farm-specific SynCom and monitor its success could be established at a local level, with the aim of having long-term effects by decreasing food prices [6]. Establishing a top-down approach using native soil microbes that are already adapted to local conditions and crops is likely to yield the most effective outcome [143]. As proposed by Delgado-Baquerizo et al., 2025, frequently monitoring soils using standardised sequencing protocols to identify changes in the microbiome, including incorporation of strains into the community and failures, is important for long-term success [144]. Taking a farm-specific approach with local monitoring would allow for minor tweaking of SynCom composition where failures of strains or unintended consequences occur. This can be linked with monitoring of available nutrients, soil conditions and crop productivity. As knowledge of the microbiomes and SynComs grows, models can be developed for better understanding of SynComs and beneficial microorganisms and their interactions with plants, soils and the environment.

## 6. Summary and Future Work

In this review, we have proposed that there should be closer and more meaningful interaction between scientists and farmers to ensure that we begin to see more direct translation of laboratory studies into the field to springboard sustainable agriculture into standard practice. Unfortunately, heavy use of agrichemicals has caused long-term harm to soils and native plant-associated microbiomes. By applying our understanding of how microbiomes become impacted over time and how they may be rebuilt into beneficial communities which interact directly with soils and crops to increase health and yields, we can help farmers become more sustainable over time. By incorporating microbes with laboratory-proven beneficial activities, including biocontrol and biopesticides that are targeted to the crop and field, reliance on agrichemicals can be reduced and soil fertility increased. Future work should begin with partnerships between scientists and farmers to identify key needs on site and the best application techniques to suit the farmer, crop and bioactivities required.

## Figures and Tables

**Table 3:** Comparison of SynCom design strategies.

Feature	Top-Down Approach	Bottom-Up Approach
Starting Point	Natural, complex microbiome from target crop or environment. Typically starts with 50–200+ species, refined/simplified to 10–30 species	Collection of individual axenic microbial isolates each with defined functional traits. Typically composed of 3–10 but can be up to 15 species
Design Principle	Holistic/ecological mindset: starting with evolved microbiome, simplify to reduce complexity, refine while maintaining desired performance characteristics	Reductionist/engineering mindset: Rationally assembled by combining species with desired/complementary functionalities
Knowledge Requirements	Moderate: requires knowledge of microbiome profile; strain isolation methodology; basic functional capacity	High: requires extensive knowledge of individual strain functionality/metabolic capacity; predictive modelling; synthetic biology; co-culture assembly
Selection Criteria	Emergent community functionality (disease suppression; P solubilisation, etc.)	Presence of specific metabolic traits/pathways or plant-growth-promotion genes
Predictability	Lower: success based on emergent properties rather than individual strain dynamics; difficult to model	Higher: precise control over species/strains: based on modelled interactions
Functional Redundancy	Natural redundancy: naturally co-evolved, may be higher than necessary but initially not defined; some microbes maybe inhibitory to others	Designed redundancy: to include 2–3 strains for each required function; balanced redundancy versus competition
Reproducibility	Moderate: high variability between natural samples	High: highly reproducible across labs/batches
Main Advantage	Co-evolved, capturing emergent properties and robustness	Enables mechanistic study of specific interactions that enables clear cause–effect relationships
Main Disadvantage	Hard to identify minimal functional unit/number of individual species required and their contribution(s)	Often lacks long-term stability and resilience
Cost Considerations	Higher per species costs: greater number of species/strains to isolate and characterise; complex production; labour intensive	Lower per species costs: fewer species/strains to characterise; simpler production; expertise intensive
Success Rate	Moderate: higher success when crop and environmental conditions are similar to source; lower when differing	Variable: high success when problem is well defined; lower when interactions are complex

## Data Availability

No new data were created or analysed in this study. Data sharing is not applicable to this article.
